# A *Schizophyllum commune* fungus ball in a lung cancer cavity: a case report

**DOI:** 10.1186/s12879-021-06739-8

**Published:** 2021-10-09

**Authors:** Naoya Itoh, Nana Akazawa, Hiromi Murakami, Yuichi Ishibana, Yusuke Takahashi, Waki Hosoda, Takashi Yaguchi, Katsuhiko Kamei

**Affiliations:** 1 Division of Infectious Diseases, Aichi Cancer Center Hospital, 1-1 Kanokoden, Chikusa-ku, Nagoya, Aichi 464-8681 Japan; 2 Collaborative Chairs Emerging and Reemerging Infectious Diseases, National Center for Global Health and Medicine, Graduate School of Medicine, Tohoku University, 2-1 Seiryo-machi, Aoba-ku, Sendai, Miyagi 980-8575 Japan; 3 Department of Thoracic Surgery, Aichi Cancer Center Hospital, 1-1 Kanokoden, Chikusa-ku, Nagoya, Aichi 464-8681 Japan; 4 Department of Pathology and Molecular Diagnostics, Aichi Cancer Center Hospital, 1-1 Kanokoden, Chikusa-ku, Nagoya, Aichi 464-8681 Japan; 5 Medical Mycology Research Center, Chiba University, 1-8-1 Inohana, Chiba, 260-8673 Japan

**Keywords:** *Schizophyllum commune*, Fungal ball, Internal transcribed spacer sequencing, Lung cancer cavity

## Abstract

**Background:**

*Schizophyllum commune* is a basidiomycete that lives in the environment and can cause infections, mainly those of the respiratory system. Although *S. commune* is increasingly reported as a cause of allergic bronchopulmonary mycosis and sinusitis, cases of fungal ball formation are extremely uncommon. Identification of *S. commune* is difficult using routine mycological diagnostic methods, and in clinically suspicious cases, internal transcribed spacer sequencing should be used for diagnosis. Here, we report a first case of lung cancer with a fungal ball formation of *S. commune*, confirmed by analyzing the internal transcribed spacer.

**Case presentation:**

A 76-year-old man with diabetes and hypertension was admitted to the hospital with a chief complaint of hemosputum, which he had for about 19 months. A computed tomography image of the patient’s chest showed a cavity and internal nodule in the left upper lobe of his lung. A left upper lobectomy was performed, and histopathological examination revealed squamous cell carcinoma of the lung and a fungal ball. The isolate from the surgical specimen was identified as *S. commune* by analyzing the internal transcribed spacer. The patient had no recurrence of the infection during 5 months of follow-up.

**Conclusions:**

Only three cases of lung fungal balls caused by *S. commune* have been previously reported, and this is the first case of lung cancer cavity with a fungal ball formation. In cases of fungal ball formation in the lung, *S. commune* should be considered a possible causative microorganism.

## Background

*Schizophyllum commune* is an environmental basidiomycete that is widely distributed in nature and grows well on rotting wood and other plants [1]. It belongs to the phylum Basidiomycota, subphylum Agaricomycotina, and order Agaricales, which includes the fungi called mushrooms [2]. Identification of *S. commune* is problematic because it is most often cultured as sterile, cottony white colonies without spore formation [3, 4]. *S. commune* is characterized by clamp connections, hyphal spicules, and formation of basidiocarps with basidiospores [4, 5]. However, unlike dikaryotic isolates, monokaryotic isolates do not show characteristic spicules or clamp connections and cannot be identified using phenotypic methods; thus, genetic sequencing is required for identification [5]. The internal transcribed spacer (ITS) regions of fungal ribosomal DNA (rDNA) are highly variable sequences of preeminent concern in identifying fungal species through polymerase chain reaction (PCR) analysis [6].

*Schizophyllum commune* is rarely involved in human disease, but it is the most common basidiomycete among the filamentous fungi to cause infections in humans [1]. This fungus causes a wide range of clinical manifestations, from allergic reactions to invasive infections, but it is primarily responsible for infections of the respiratory system. Bronchopulmonary infections and sinusitis account for more than 90% of reported cases worldwide [7]. These localizations are consistent with natural airborne transmission through inhalation of basidiospores released into the atmosphere. The infection may remain localized or spread from the original site to other tissues and organs depending on factors such as the immune status of the host, deviation of the nasal septum, use of corticosteroid therapy, and duration of exposure to the spores [8].

Fungal balls of the lung are masses of fungal mycelium growing in existing cavities. They are found in patients with underlying lung diseases such as tuberculosis, a history of systemic fungal infections, recurrent bacterial pneumonia, lung abscesses, sarcoidosis, and cavitated squamous cell lung cancer [4, 9]. In most cases, species of *Aspergillus*—most commonly *A. fumigatus*—or *Scedosporium apiospermum* (*Pseudallescheria boydii*) are involved [4]. Cases of pulmonary fungal ball formation caused by *S. commune* are extremely rare [4, 8, 10]. We report the first case of lung cancer cavity lesion with a fungal ball formation of *S. commune*. Sequencing analysis of the ITS was used to identify the microorganism.

## Case presentation

A 76-year-old Japanese man with diabetes and hypertension was admitted to our hospital with hemosputum, a symptom which he had for about 19 months. The patient had gone to another hospital 19 months earlier for hemosputum and was diagnosed with a nodule (42 × 24 mm) in the left upper lobe of his lung after undergoing a chest computed tomography scan. The chest scan from 18 months earlier showed that the nodule was associated with cavitation. A bronchoscopy was performed, but the cause of the hemosputum was not found. The patient was referred and then admitted to our hospital for diagnosis because the hemosputum persisted and lung cancer was suspected. The patient’s medical interview conducted at our hospital revealed a history of a 3-month stay in Chiang Mai, Thailand, four years previously. There was no other history of dust or tuberculosis exposure, nor any family history, any pets, or any history of gardening or mountain hiking.

**Fig. 1:**
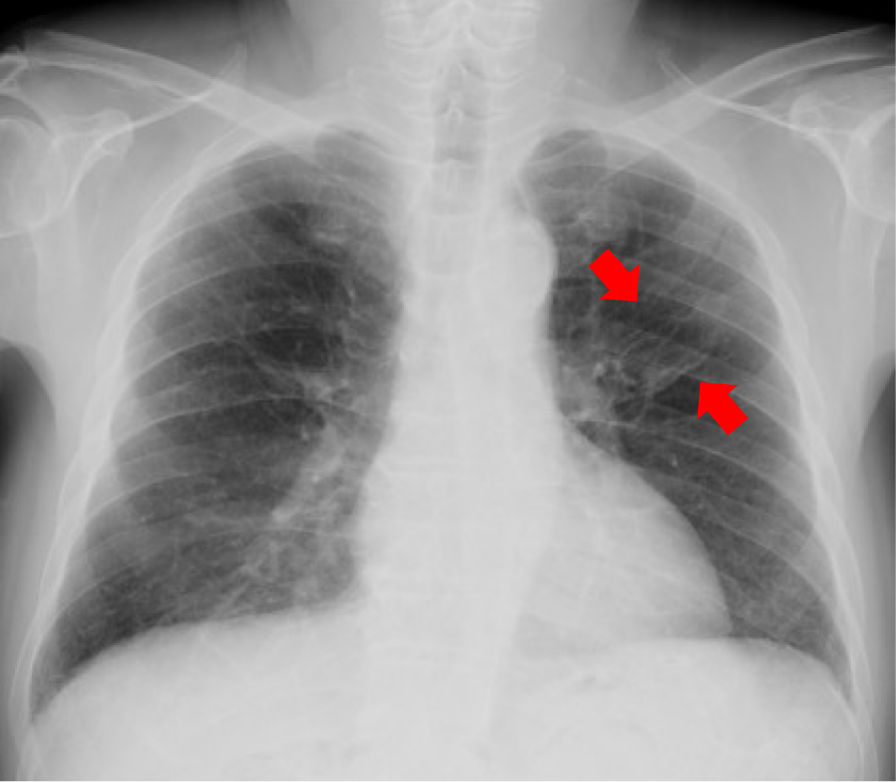
Chest radiographs showing the cavitary lesion (red arrows) in the left middle lung field

**Fig. 2:**
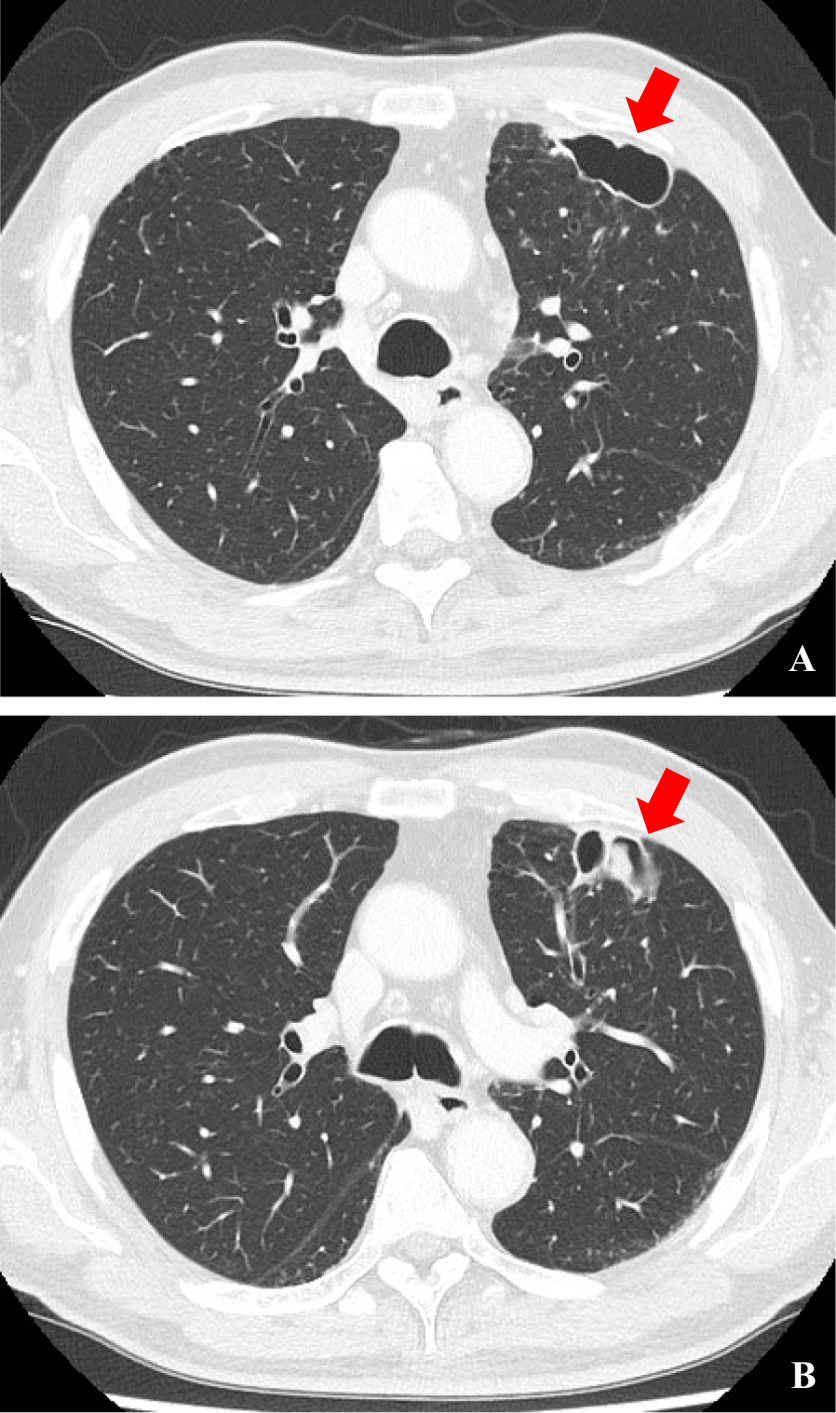
Computed tomography scan of the thorax. The left upper lobe of the lung has a cavity (**A**, **B**) and internal nodule (**B**); these are indicated using red arrows

On examination, the patient appeared well. His temperature was 36.2 °C, heart rate was 76 bpm and regular, blood pressure was 147/81 mmHg, and oxygen saturation was 97% in room air. His physical examination findings were unremarkable. Laboratory investigations revealed a white blood cell count of 7090/μL (neutrophil count 4552/μL, eosinophil count 177/μL), hemoglobin level of 18.4 g/dL, C-reactive protein level of 0.02 mg/dL (normal < 0.30 mg/dL), and hemoglobin A1c level of 7.5%. His chest radiographs showed a cavitary lesion in the left middle lung field (Fig. 1). His chest scan showed a cavity (42 × 24 mm) in the left upper lobe with an internal nodule (13 × 11 mm) (Fig. 2). In addition to lung cancer, aspergilloma and tuberculosis were considered differential diagnoses. Serum beta-D-glucan and *Aspergillus* antibody tests were negative. Bacterial culture of his sputum showed oral commensals and methicillin-resistant *Staphylococcus aureus*. The culture and smear of three series of sputum for anti-acid bacteria were negative.

**Fig. 3:**
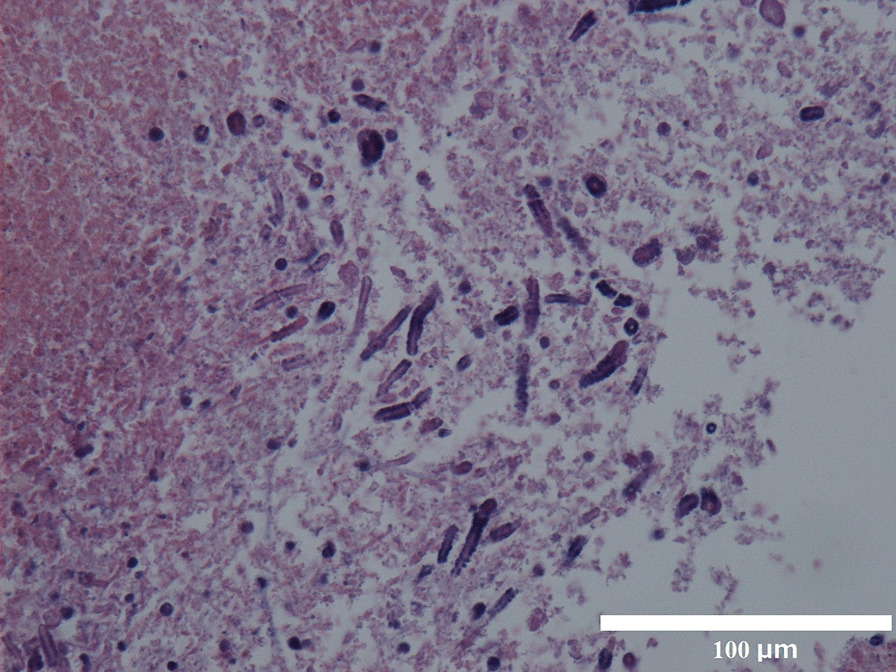
Histologic section of a specimen obtained from the fungus ball showing septate fungal hyphae (hematoxylin and eosin staining) (magnification× 400, 300 dpi). (This image was acquired and captured using a Nikon eclipse 55i microscope (Nikon, Japan) and a Nikon Digital Color Camera Sight DS-Fi-1 (Nikon, Japan)

**Fig. 4:**
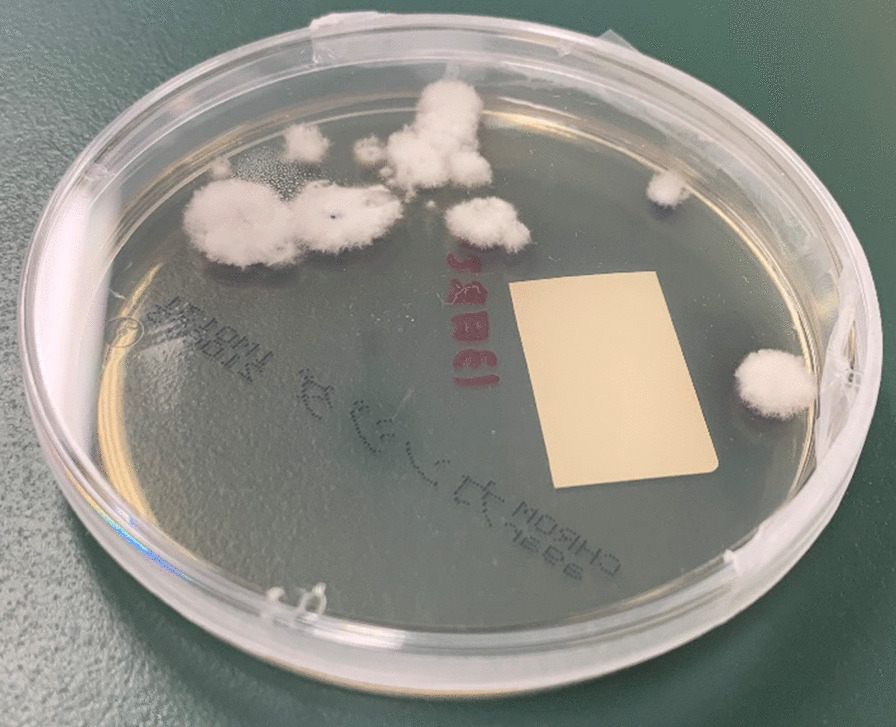
Multiple white, woolly colonies of *Schizophyllum commune* after a 7-day incubation on a CHROMagar Candida plate

**Fig. 5:**
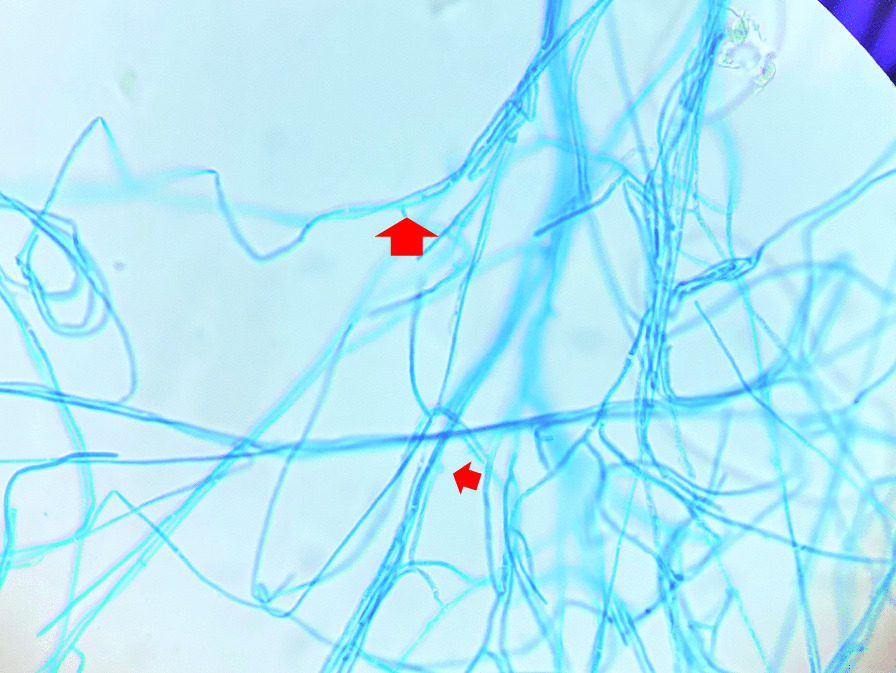
Lactophenol cotton blue mount of slide culture on CHROMagar of *Schizophyllum commune*. The image shows the hyaline hyphae with clamp connections (small arrow) and spicules (large arrow). No conidia are visible. Photomicrograph obtained at ×400 magnification (300 dpi)

A thoracoscopic left upper lobectomy was performed on the patient, and he was discharged on the second postoperative day. Histopathological examination of the cavitary lesion in the left upper lobe revealed squamous cell carcinoma, and the nodule in the cyst was identified as a fungal ball with numerous filamentous fungi on Hematoxylin and eosin staining (Fig. 3). Lung specimens were cultured aerobically at 35 °C on a CHROMagar Candida plate (Kanto Chemical Co, Inc., Tokyo, Japan), sheep blood agar (Nissui Pharmaceutical Co., Ltd., Tokyo, Japan), and chocolate agar EX II (Nissui Pharmaceutical Co., Ltd., Tokyo, Japan). No growth was observed at 48 h, and the incubation was continued at 25 °C. At 72 h, small white colonies developed (Fig. 4). Lactophenol cotton blue mounts of slide cultures on CHROMagar of the isolated fungus showed hyaline hyphae with clamp connections and spicules, but no conidia (Fig. 5).

Since we were not able to identify the fungal species at our institution, we asked the Medical Mycology Research Center (MMRC), Chiba University to identify the fungi. The ITS sequencing of the isolated fungus was performed in MMRC. Based on the Basic Local Alignment Search Tool (BLAST) search of the sequence on the ITS region of the rRNA gene of the isolated fungus, the homology of the standard strain *S. commune* CBS 124811 (GenBank Accession No.: MH863418) was 99.8% (617/618 bp) [11]. Thus, we identified the isolate as *S. commune* on phylogeny and deposited it as IFM 67,107 at MMRC, Chiba University, through the National Bio-resource Project, Japan. The final diagnosis was a fungal ball caused by *S. commune* in the cavity of the lung cancer. The patient has not experienced recurrence of symptoms in 5 months of follow-up.

## Discussion and conclusions

We report the first case of lung cancer cavity with a fungal ball formation of *S. commune* identified by analyzing the ITS.

*S. commune* is a basidiomycete characterized by the formation of clamp connections, hyphal spicules, and basidiocarps with basidiospores [8]. The fungus is an opportunistic pathogen that can cause a wide range of clinical manifestations, including sinusitis, allergic bronchopulmonary mycosis, eye, ear, and skin infections, abscesses, and fungemia; however, human infections are rarely reported [1]. According to a previous worldwide study of the 71 cases of *S. commune* reported, 45 (63%) were bronchopulmonary and 22 (31%) were sinusitis [8]. There have been only three previous reports of fungal ball formation by *S. commune* [4, 8, 10], and ours is the first case reported of it forming in a lung cancer cavity. *S. commune* is distributed all over the world [8], and the patient’s history in Chiang Mai was not considered a special risk. However, it has been noted that infections caused by *S. commune* are underestimated [8]. *S. commune* is cultured according to a routine laboratory culture method as described in our case. Identification based solely on macro-microscopic characteristics of cultured colonies is challenging in practice because *S. commune* generally does not form spores and grows woolly, whitish, and sterile colonies [1]. Long incubation times are required for its characteristic macroscopic structures to develop, making cultured colonies impractical for clinical diagnosis [1]. Since the reference spectral library of matrix-assisted laser desorption ionization-time of flight mass spectrometry (MALDI-TOF MS) is still incomplete, timely use of molecular techniques should be considered the gold standard for diagnosing *S. commune* in clinically suspicious cases [12]. The infecting organism in our case was finally identified by analyzing the ITS, and a case of fungal ball formation within the lung cancer cavity caused by *S. commune* was diagnosed.

Most cases of fungal balls are associated with *Aspergillus* [4]. Certain imaging features—such as the air crescent sign, meniscus sign, and ball-in-hole sign—are associated with the presence of a fungal ball, which results in a collection of air that is shaped like a crescent and that separates the wall of the cavity from an internal mass [13–15]. These imaging findings have been reported to be associated with a variety of other diseases, and differential diagnosis can be difficult. Other causes of intracavitary masses surrounded by a crescent of air include other fungi, pulmonary hydatid cyst, Rasmussen aneurysm, pulmonary gangrene, intracavitary clot, textiloma, lung cancer, metastases, and teratoma [16]. In our case, the possibility of lung cancer, aspergilloma, or tuberculosis was considered preoperatively, but the nodule was finally diagnosed as a fungal ball of *S. commune* that had developed in the cavity of the lung cancer. There are very few reports of fungal ball formation within the lung cancer cavity, and all are due to aspergilloma [9, 17, 18]. The possibility of cell–cell interactions (i.e., between the cancer and *Aspergillus*) and their impact on the growth, differentiation, and overall invasiveness of these cancers has not been studied [19]. In our case, the relationship between lung cancer and *S. commune* is unclear, but it was shown that *S. commune* can form a fungus ball in lung cancer cavities. Therefore, *S. commune* infection should be considered one of the causes in cases with pulmonary fungal balls.

Symptoms of and risk factors for fungal balls caused by *S. commune* are still unknown due to the scarcity of reports. However, according to previous reports [4, 8, 10] and ours, three out of four patients had blood sputum and hemoptysis, and three out of four had diabetes mellitus (Table 1). Only one of the four patients had no history or symptoms of diabetes. Since our patient had diabetes mellitus, as did patients in previous reports, diabetes was considered a risk factor.

**Table 1:** Summary of published cases of fungal balls due to *Schizophyllum commune*

Author, country, year, reference	Age (years), sex	Symptoms	Underlying condition	Diagnostic method	Susceptibility MEC or MIC (μg/mL)	Treatment	Outcome
Sigler, Canada, 1995, [4]	53, F	Cough Hemoptysis	TB, DM	Morphotype, Analyzing ITS	ND	Lobectomy	Survived
Chowdhary, India, 2013, [8]	42, M	Hemoptysis	TB, DM	Morphotype, Immunodiffusion test, Specific IgE, Skin test, Analyzing ITS	ITC < 0.06–0.125; VRC 0.5–1; POS 0.015–0.125; AMB 0.5–1; CAS 0.125–0.5; AFG 0.125–0.5; MFG 0.125–0.5	Systemic glucocorticoids ITC, 4 months	Survived
Sakaguchi, Japan, 2018, [10]	58, F	None	None	Morphotype, Analyzing ITS	ND	Lobectomy	Survived
Present case	76, M	Hemosputum	Lung cancer, DM, HT	Morphotype, Analyzing ITS	ND	Lobectomy	Survived

MEC, Minimum effective concentrations; MIC, minimum inhibitory concentration; F, female; M, male; ND, not done; DM, diabetes mellitus; TB, tuberculosis; HT, hypertension; AMB, amphotericin B; IgE, immunoglobin E; ITC, itraconazole; FLC, fluconazole; VRC, voriconazole; MFG, micafungin; POS, posaconazole; CAS,

The role of surgery and antifungal therapy in cases of a fungal ball caused by *S. commune* remains unclear due to the rarity of reports on these clinical manifestations and limited experience in their management. Of the four cases reported to date, including our own, three were treated with lobectomy alone without antifungal therapy; one patient was treated with itraconazole for 4 months. Data on in vitro antifungal susceptibility testing of *S. commune* are scarce. Previous reports have shown that antifungal susceptibility testing of itraconazole, voriconazole, and amphotericin B resulted in very low minimum inhibitory concentrations [5, 20–24]. In this case, although drug susceptibility testing was not performed, considering that the condition was similar to simple pulmonary aspergilloma, surgical treatment was appropriate. In addition, no recurrence was observed at the 5-month postoperative follow-up. The disease recurrence after lobectomy remains unknown, and whether the same treatment plan as that for aspergilloma is appropriate requires further studies. However, our patient is being carefully followed up in an outpatient clinic, and no recurrence has been observed at 5 months after surgery.

In conclusion, we report the first case of lung cancer cavity lesion with a fungal ball formation of *S. commune*. The case was diagnosed by analyzing the ITS, and the patient underwent lobectomy, with no sign of recurrent infection 5 months after the surgery. In cases of fungal ball formation in the lung, *S. commune* should be considered a possible causative microorganism.

## Data Availability

The data used and/or analyzed during the current study are available from the corresponding author on reasonable request.

## Abbreviations

BLAST: Basic Local Alignment Search Tool
MALDI-TOF MS: Matrix-assisted laser desorption ionization-time of flight mass spectrometry
ITS: Internal transcribed spacer
MMRC: Medical Mycology Research Center
PCR: Polymerase chain reaction
rDNA: Recombinant DNA
